# Construction of CF_2_ moieties in FDA-approved drugs (2016–2025): industrial routes, mechanisms, and scale-up considerations

**DOI:** 10.3762/bjoc.22.91

**Published:** 2026-08-04

**Authors:** Wenyan Yao, Huanhuan Zhang, Xiaolong Yang, Feng Liu

**Affiliations:** 1 Shaanxi Institute of International Trade & Commerce, Xianyang, 712046, China; 2 Shaanxi Buchang Pharmaceutical Co., Ltd., Xi'an, 710075, China

**Keywords:** difluoromethylene group (-CF_2_-), difluoromethyl group (-CF_2_H), FDA-approved drugs, fluorination methods, process scale-up

## Abstract

The incorporation of CF_2_ moieties into drug molecules offers unique advantages in improving metabolic stability and target affinity, making their efficient construction a major focus in pharmaceutical process chemistry. This review systematically examines the synthetic processes of 12 FDA-approved CF_2_-containing drugs from 2016 to 2025, categorizing them according to the chemical environment of the CF_2_ group (alkyl-CF_2_, heteroaryl-CF_2_, and ArO-CF_2_). For each drug, the industrial routes for CF_2_ construction and the reasons for eliminating alternative routes are analyzed. Methods for CF_2_ formation are classified into two major strategies: fluorinating reagent-based approaches and building block-based approaches. Key process steps and scale-up data are summarized in Table 1. The article systematically discusses four types of reaction mechanisms – nucleophilic fluorination, electrophilic fluorination, radical pathways, and carbene insertion – and evaluates their applicability in industrial production. The analysis shows that for alkyl-CF_2_ groups, the industry strongly favors preformed CF_2_ building blocks or indirect fluorination strategies to avoid hazardous reagents and harsh conditions. Continuous flow technology using SF_4_ has emerged as an important complement to traditional fluorination methods. For heteroaryl-CF_2_ groups, metal-catalyzed direct introduction of the CF_2_ unit still faces challenges for industrial application, whereas the difluorocarbene route for ArO-CF_2_ has been validated. This review provides a reference for process development of new CF_2_-containing drugs, from strategic design to scale-up evaluation.

## Introduction

In recent years, the CF_2_ group has demonstrated unique advantages in drug discovery and clinical therapy. The introduction of fluorine-containing groups such as CF_2_ into drug molecules can significantly alter their properties, including metabolic stability, lipophilicity, conformation, and hydrogen-bonding capacity [1]. For example, fluorine substitution on sugar moieties can modify nucleoside conformations, while aromatic C–F groups can influence arene–arene interactions by altering the electronic properties of the aromatic system. Such property modifications can block unnecessary oxidative drug metabolism, thereby improving metabolic stability and enhancing affinity with target enzymes, leading to increased drug efficacy [2]. With the continuous discovery and application of fluorine-containing drugs, the stepwise introduction of fluorine from fluorite – the primary source of fluorine in the Earth's crust – into drug molecules in an economically viable and environmentally friendly manner has become a critically important research area. The supply chain of fluorinated organic molecular raw materials has attracted increasing attention [3]. In particular, the construction of CF_2_ moieties remains a research hotspot. Recent advances in this area include the synthesis of aza-heterocyclic compounds bearing in-ring CF_2_ fragments, as reviewed in 2024 [4]. In 2025, Sun et al. [5] systematically reviewed recent methods for constructing CF_2_ groups, categorizing them by reaction mechanism (radical difluoromethylation, transition-metal-catalyzed difluoromethylation, nucleophilic and electrophilic difluoromethylation). Efficiently introducing CF_2_ groups into drug molecules not only facilitates the rapid discovery of candidate compounds in early-stage drug discovery but also enables the economical, environmentally friendly, and reliable large-scale production of active pharmaceutical ingredient (API) samples during clinical and commercial manufacturing phases.

The term "CF_2_" structural unit discussed in this review broadly refers to fragments in drug molecules containing a *gem*-difluoromethylene group (–CF_2_–) or a difluoromethyl group (–CF_2_H), as well as derivatives such as –CF_2_Cl. In addition, molecules containing longer perfluoroalkyl chains that incorporate CF_2_ fragments, such as 1-(perfluorohexyl)octane, are also included in the scope of this review due to the presence of the CF_2_ unit within their structures. Compounds bearing monofluoro or trifluoromethyl (–CF_3_) groups are excluded, as their fluorination strategies differ fundamentally from the CF_2_ incorporation methods discussed herein.

**Figure 1:**
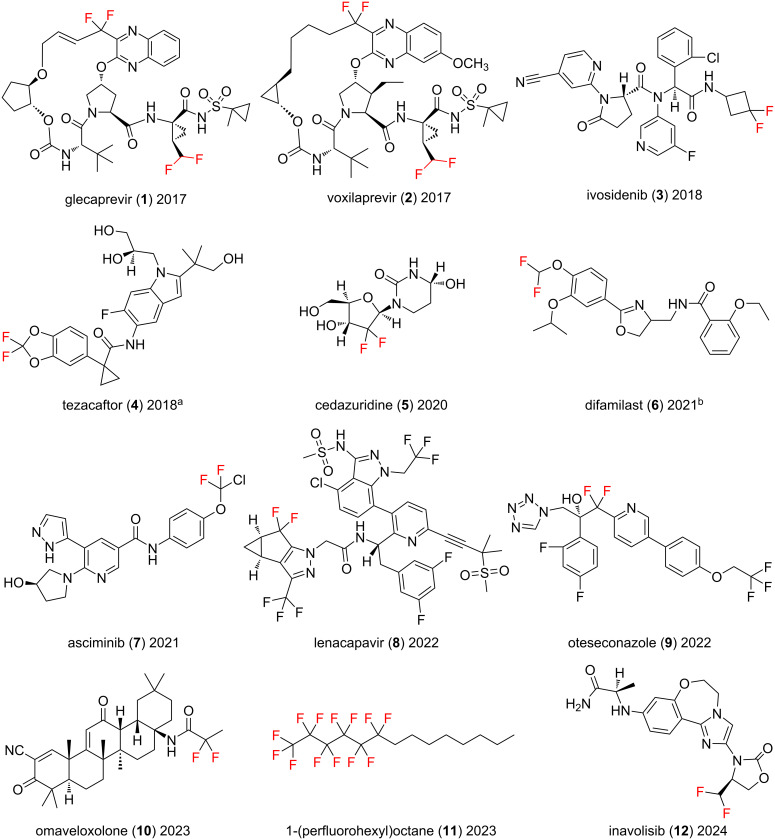
FDA-approved drugs containing CF_2_ groups (2016–2025). a) For tezacaftor (**4**), the 2018 approval year shown refers to its first FDA approval as a new molecular entity in combination with ivacaftor (symdeko); subsequent approvals in 2019 (Trikafta) and 2024 (Alyftrek) represent new fixed-dose combinations. b) Difamilast (**6**) was first approved by the Japanese PMDA in 2021 and subsequently received FDA approval in 2026 (outside the 2016–2025 timeframe). It is included here for its structural and synthetic relevance.

This article reviews the FDA-approved drugs containing CF_2_ groups from 2016 to 2025, totaling 12 compounds (Figure 1). Difamilast (**6**) was approved in Japan in 2021, and its FDA approval followed in 2026, which falls slightly outside the designated timeframe of this review. Nevertheless, owing to its representative ArO-CF_2_ structure and the instructive value of its synthetic approach, it has been included in the discussion as a special case. Although the search window for this review was set as 2016–2025 (a ten-year period), a comprehensive search revealed that no CF_2_-containing drugs received FDA approval in 2016 or 2025. Consequently, the drugs shown in Figure 1 are concentrated in the 2017–2024 timeframe.

The review summarizes the synthetic methods used to construct the CF_2_ moieties in these 12 drugs, compares the advantages and disadvantages of various approaches, and highlights efficient, industrially viable routes and methods suitable for large-scale production, aiming to provide a reference for the research, development, and manufacturing of new CF_2_-containing drugs. In general, there are two strategies for introducing CF_2_ groups: reacting organic fluorinating reagents with functional groups in drug intermediates to obtain the CF_2_ moiety (fluorine reagent-based approach), and using starting materials that already contain CF_2_ or analogous groups for construction (building block-based approach). The drugs are categorized according to the chemical environment of the CF_2_ group (alkyl-CF_2_, heteroaryl-CF_2_, and ArO–CF_2_). Their synthetic methods are presented in section 1 following the same classification, while the mechanistic discussion is reserved for section 2. This review is primarily intended for researchers engaged in process chemistry. It focuses on sorting out the synthetic feasibility, the evolution of the reported routes, and the key advantages of the ultimately adopted industrial routes for approved CF_2_-containing drugs. The aim is to provide a reference for the synthetic route design and process scale-up of future CF_2_-containing molecules.

To help readers distinguish between routes that have been demonstrated at production scale and those reported only at the laboratory level, the following visual conventions are adopted in the schemes. Industrial steps or routes for which literature-reported data indicate scale-up to at least kilogram quantities are marked with bold arrows and highlighted in blue. Promising scale-up technologies, such as continuous-flow methods, are highlighted in the same manner. Other reported routes retain their conventional formatting.

## Review

### 1 Methods for constructing CF_2_ groups in FDA-approved CF_2_-containing drugs (2016–2025)

The 12 drugs shown in Figure 1 are classified into three types according to the chemical environment of the CF_2_ group: alkyl-CF_2_, heteroaryl-CF_2_, and ArO-CF_2_. Glecaprevir (**1**) and voxilaprevir (**2**) have similar structures, both containing an heteroaryl-CF_2_ and an alkyl-CF_2_ group. The synthetic methods are summarized below according to these three structural types. Within each subsection, the drugs are presented in chronological order based on their year of approval.

In evaluating the reported routes, multiple dimensions were taken into account, including reagent safety, cost, scalability, step economy, and the availability of upstream fluorinated feedstocks. Comments throughout the text such as "requires hazardous reagents," "high cost," or "harsh conditions" are based on information explicitly reported in the original literature or patents. It should be noted that the final choice of an industrial route typically results from a multifactorial balance, and the description of a single route's advantages or disadvantages should not be interpreted as the sole reason for its adoption or abandonment. In this review, the term "commercially available" generally refers to industrial-scale availability.

#### 1.1 Construction strategies for alkyl–CF_2_

The most common method for constructing alkyl–CF_2_ is based on the deoxyfluorination of ketone or aldehyde carbonyl groups using organic fluorinating reagents such as diethylaminosulfur trifluoride (DAST). However, safety concerns associated with DAST have prompted the development of alternative methods that avoid direct fluorination of carbonyl groups. Most of the six drugs discussed in this section employ process-scale alternatives to DAST, such as building block approaches and continuous-flow methods using SF_4_.

**Figure 2:**
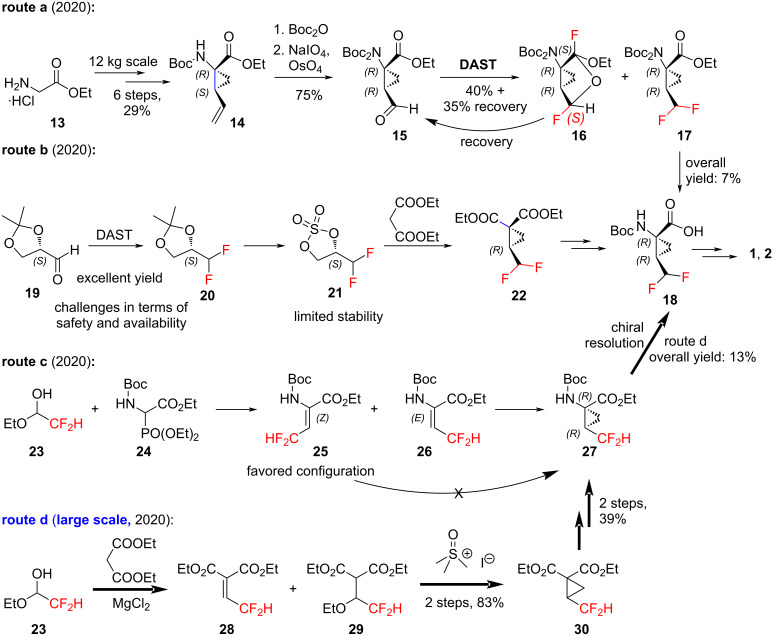
Synthesis of the alkyl–CF_2_ side chain in glecaprevir (**1**) and voxilaprevir (**2**).

**1.1.1 Glecaprevir and voxilaprevir (2017):** As shown in Figure 2, compound **18** is the key alkyl–CF_2_ side chain for the synthesis of **1** and **2**. There are four main methods for its construction: Route a [6] and route b [7] are based on fluorination of the terminal aldehyde group; route c [7] and route d [7] are building block approaches, with route d being the final industrial route. All routes above were reported in 2020 (the conversion of **13** to **14** in route a was reported in 2005 [8]).

The early route a [6] introduced the difluoromethyl group via deoxyfluorination of the aldehyde group in molecule **15** using DAST (a nucleophilic fluorination mechanism, see section 2.1.1 for details). This fluorination step was highly challenging. After a series of optimizations, the optimal process on the hundred-gram scale achieved only 40% yield, due to the formation of the five-membered ring impurity **16**, which was influenced by the three-membered ring and the ester bond in molecule **15** itself. This impurity was successfully recovered as the dithioacetal of **15** in 35% yield. Overall, although intermediate **14** had already been synthesized on a 12 kg scale (29% yield over six steps, using an inexpensive enzymatic process [8]), route a suffered from several scale-up issues: the use of OsO_4_ (highly toxic) and DAST (hazardous), the low fluorination yield, and a low overall yield of only 7% over ten steps.

Route b [7] used compound **19**, which contains an aldehyde group and a chiral carbon center, as the starting material, solving the problem of the difficult availability of the aldehyde starting material in route a. While the yield of the fluorination step was very high, DAST was still required, and the limited stability of sultone **21** hindered scale-up production.

In an alternative approach, building-block strategies for cyclopropane ring formation were developed in route c [7] and route d [7]. The starting material for both routes is the commercially available compound **23**, which is inexpensive. Compound **23** can be obtained from ethyl difluoroacetate [3] via reduction with Red-Al [9].

The Corey–Chaykovsky cyclopropanation is a powerful method for constructing cyclopropane rings. Given the stereochemistry of **18**, the key to this method lies in the construction of the *E*-olefin **26**. However, in route c, the first step, a Horner–Wadsworth–Emmons reaction, tends to give the *Z*-olefin as the product, which is unfavorable for the subsequent cyclopropane ring formation. Attempts to use chiral auxiliaries used to promote *E*-olefin formation gave extremely low yields in the synthesis of **26**. In route d, during the Knoevenagel condensation in the first step, due to the exceptionally electrophilic nature of alkylidene **28**, the reaction also produced **29**, an addition product of **28** with ethanol, as well as an addition product of **28** with water. Screening of Lewis acids revealed that the addition of magnesium chloride effectively suppressed the formation of the water adduct, and although the by-product **29** was formed in up to 90% yield, both **28** and **29** were competent substrates in the subsequent cyclopropanation, giving an 83% two-step overall yield. Subsequent steps, including selective ester hydrolysis (56% yield), Curtius rearrangement (70% yield), chiral resolution, and ester hydrolysis (40% yield over two steps), afforded the target product. Route d employs a building block approach to construct the terminal CF_2_ group on the cyclopropane ring, over eight steps, affording an overall yield of 13% on large scale.

**Figure 3:**
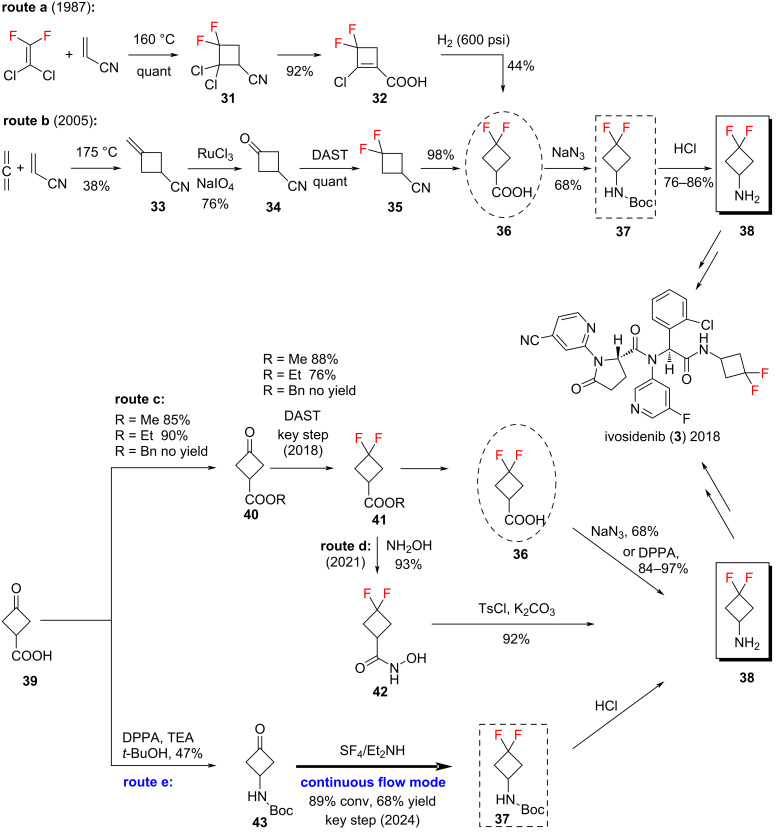
Synthesis of the difluorinated intermediate **38** for ivosidenib (**3**).

**1.1.2 Ivosidenib (2018):** As shown in Figure 3, there are five routes for the synthesis of the difluorocyclobutylamine side chain **38** in ivosidenib (**3**). Among them, route a (reported in 1987) [10] and route b (reported in 2005) [11] are both methods for constructing the four-membered ring. Routes c, d, and e use cyclobutane-containing starting materials and synthesize the target compound **38** via deoxyfluorination of a ketone carbonyl group. Route c, assembled from literature [12–14], was reported between 2013 and 2021, key CF_2_ introduction reported in 2018. Route d [12] was reported in 2021. Route e, assembled from literature [15–16], was reported between 2017 and 2024, key CF_2_ introduction reported in 2024.

In route a [10], the CF_2_ group originates from the building block 1,1-dichloro-2,2-difluoroethene. However, the first step (ring closure) and the third step (hydrogenation) require high-temperature and high-pressure conditions, respectively. Moreover, the yield of the third-step reductive dehalogenation was only 44%. In route b [11], the first step, which requires high temperature, gave only 38% yield [13], and DAST was used in the third step. In addition, both routes use acrylonitrile as a starting material, which is highly toxic and prone to explosion at elevated temperatures.

Routes c, d, and e all use 3-oxocyclobutane-1-carboxylic acid (**39**) as the starting material and prepare the key intermediate via two strategies. Routes c and d follow the sequence of fluorination followed by Curtius rearrangement, with the carboxylic acid group requiring prior esterification before fluorination. The yields for esterification and fluorination were 85% and 88% for the methyl ester [12], and 90% and 76% for the ethyl ester [13], respectively (the yield for the benzyl ester was not reported [14]). Route e proceeds via Curtius rearrangement first, followed by fluorination. It is noteworthy that a study [13] found that the yield of the difluoro acid **38** in route c decreased significantly during scale-up when using diphenyl phosphoryl azide (DPPA) for the Curtius rearrangement (the reason for the decrease was not described). Switching to the Schmidt reaction using NaN_3_ gave a yield of 68%, but the safety profile of sodium azide is far less favorable than that of DPPA. Route d also starts from compound **39** and proceeds through fluorination of **40** to **41** using DAST, followed by conversion of **41** to **38** in two steps with a combined yield of 85% [12]. This route avoids the use of azide reagents.

The continuous-flow technology has become a key means of reducing operational risks associated with handling hazardous chemicals. Explosive, toxic, and corrosive intermediates and by-products (such as hydrofluoric acid) can be quenched online immediately after the reaction, thereby significantly enhancing safety. In route e, during the fluorination of **43**, an SF_4_/Et_2_NH continuous-flow synthesis was employed for the deoxyfluorination of the ketone [16], with a yield of 68% (89% conversion). This approach effectively generates DAST in situ and uses it immediately. Furthermore, this strategy is not only applicable to four-membered ring ketones but also shows good applicability to five-and six-membered ring ketone substrates. It is worth noting that, in contrast, the continuous-flow method using SF_4_ alone can achieve fluorination of alcohols, aldehydes, and carboxylic acids, but shows lower applicability to ketone compounds [17]. In route e, compound **39** was transformed into **43** with DPPA in 47% yield [15]. Replacing the Boc group in **43** with Cbz gave a Curtius rearrangement yield of 25% and a DAST fluorination yield of 69% [18].

In summary, regardless of the strategy adopted, two key points are most critical. First, the fluorination step should avoid the direct use of the DAST reagent; for example, the continuous flow approach used in route e can be adopted. Second, the conversion of the carboxy group to an amino group should avoid the use of azide reagents whenever possible. When DPPA performs poorly, route e provides a reliable alternative. The above routes and yield data are all based on reports at gram to hundred-gram scales. For an FDA-approved drug such as ivosidenib, economically viable manufacturing processes must have been established, at least internally, by the relevant pharmaceutical companies. These processes may not be fully disclosed in the open literature or patents, and improved routes may of course be developed in the future. It should be noted that the five routes summarized above span from 1987 to 2024. The earlier routes were developed under the technological constraints of their time, while later routes benefitting from advances in continuous-flow technology and building-block strategies. Rather than evaluating routes from different eras against identical criteria, this comparison is intended to illustrate the evolution of synthetic approaches and to provide a useful reference for the design and scale-up of synthetic routes to future CF_2_-containing drug candidates.

Finally, it should be particularly noted that under forced photodegradation conditions, the difluoromethylene group of ivosidenib (**3**) is susceptible to defluorination and hydrogenation, leading to conversion to a methylene group [19]. Therefore, for drugs containing similar difluoromethylene structural units, attention must be paid to the influence of light exposure during both synthesis and storage.

**Figure 4:**
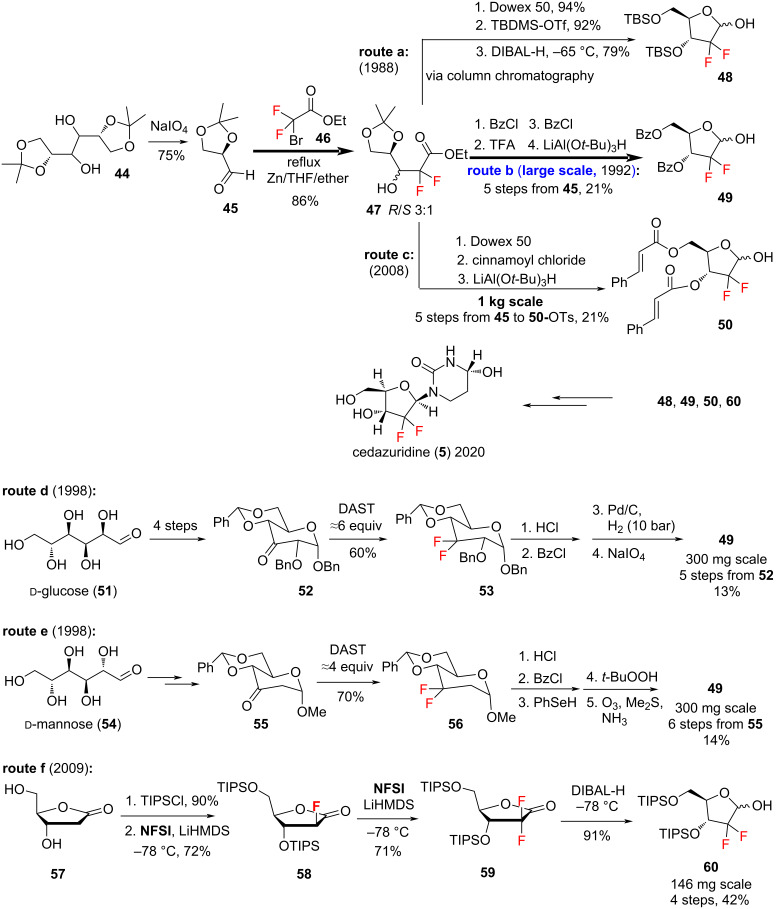
Synthesis of the difluorinated intermediate for cedazuridine (**5**).

**1.1.3 Cedazuridine (2020):** As shown in Figure 4, the key intermediates for the synthesis of cedazuridine (**5**) are the 2-deoxydifluoro sugar hydroxy compounds **48**–**50** and **60**, which bear different protecting groups and are also key intermediates in the synthesis of gemcitabine. The synthetic challenge lies in the simultaneous construction of the CF_2_ group and the chiral furanose ring. Among the six routes shown in Figure 4, the methods for introducing the CF_2_ group include the building-block approach, the DAST method and electrophilic fluorination. While the synthesis of the chiral furanose ring employs intramolecular transesterification, conversion of a pyranose ring to a furanose ring, and the building block approach. Route b is the commercial route.

Early route a (reported in 1988) [20] provided a synthetic method for **48**. Compound **45** was subjected to a Reformatsky reaction with **46** to obtain a CF_2_-containing C5 backbone (stereoselectivity *R*/*S* = 3:1). The *R* isomer was isolated by silica gel column chromatography (65% yield), followed by stirring with Dowex 50W-X12 resin at room temperature for 4 days to form the furanose ring (94% yield). Subsequent TBS protection (92% yield) and diisobutylaluminium hydride (DIBAL-H) reduction at −65 °C (79% yield) afforded **48**.

Subsequently, researchers developed a commercial route b (reported in 1992) [21] that removes chiral isomers by recrystallization. The specific steps are as follows: Bz protection of **47** (96% yield), trifluoroacetic acid (TFA) deprotection followed by azeotropic distillation to effect ring closure (quantitative yield), second Bz protection and recrystallization to remove chiral isomers (26% yield, 100% ee), and finally reduction of the ester carbonyl to the alcohol **49** using lithium tri-*tert*-butoxyaluminium hydride (LTBA) at 10 °C (quantitative yield). From **45** to **49**, the five-step process gives an overall yield of 21%. It is worth noting that, due to the presence of the difluoromethylene group, no pyranose ring by-product was detected during the ring-closing step.

Route c (reported in 2008) [22] uses a cinnamoyl group as a protecting group in place of the Bz group in route b, and also removes chiral isomers by recrystallization. After obtaining intermediate **50**, it is converted to **50**-OTs using *p*-toluenesulfonyl chloride (TsCl). Starting from **45** to **50**-OTs, the process proceeds in 5 steps with an overall yield of 21% on a 1 kg scale.

In the above three routes, the introduction of the difluoromethylene group relies on the asymmetric Reformatsky reaction induced by the chiral substrate **45**. The stereoselectivity of the product has always been a challenge in this type of synthesis, and developing more efficient asymmetric Reformatsky methods would undoubtedly improve production efficiency. Lv et al. [23] reported a chromium-catalyzed asymmetric Reformatsky reaction, finding that both BrCF_2_COOEt (**46**) and ClCF_2_COOEt can react with aliphatic or aromatic aldehydes under this reaction system to afford chiral alcohols in moderate to high yields with high stereoselectivity. Notably, BrCF_2_COOEt requires blue light irradiation to react, whereas ClCF_2_COOEt proceeds smoothly under conventional mild conditions without the need for photochemical reactions. This strategy offers a new approach for the synthesis of **47**.

Routes d, e, and f all start from chiral raw materials, thus completely avoiding the formation of chiral isomers. Routes d and e (reported in 1998) [24] both employ the DAST reagent to introduce the difluoromethylene group. Route d proceeds from **52** to **49** in 5 steps, involving a high-pressure hydrogenation step, with an overall yield of 13% (9 steps if starting from ᴅ-glucose (**51**). Route e proceeds from **55** to **49** in 6 steps with an overall yield of 14%; starting from ᴅ-mannose (**54**), the route is even longer. Although both routes were conducted on a 300 mg scale and have low overall yields, they are the only synthetic routes reported in the early literature that avoid introducing chiral isomers. In particular, they pioneered two synthetic methods that convert pyranose ring intermediates into furanose rings, providing valuable references for the synthesis of the 2-deoxydifluorosugar ring system.

Route f (reported in 2009) [25] starts from **57**, which contains two pre-existing chiral centers, and proceeds in four steps to afford **60**, with the last three steps all requiring a temperature of −78 °C. After protection of **57**, two sequential electrophilic fluorinations at the 2-position of the sugar ring are carried out under lithium bis(trimethylsilyl)amide (LiHMDS) and *N*-fluorobenzenesulfonimide (NFSI) conditions, via an electrophilic fluorination mechanism, to give **58** and **59** in 72% and 71% yield over the two steps, respectively. The authors speculated that the key to this electrophilic fluorination lies in the use of bulky silyl protecting groups at the 3- and 5-positions of lactone **57**. Upon deprotonation of C2 by LiHMDS, the resulting enolate potentially faces a competing elimination pathway. A sufficiently bulky silyl group (e.g., triisopropylsilyl (TIPS) or *tert*-butyldimethylsilyl (TBDMS)) locks the ring into a C3-*endo* pucker, forcing the C3–O bond into a pseudo-equatorial orientation. This geometry prevents the antiperiplanar alignment needed for elimination. As a result, elimination of the siloxide is suppressed, and the enolate is efficiently trapped by the electrophilic fluorinating reagent. Consistent with this rationale, TIPS afforded the fluorinated product in 72% yield, compared to 58% with TBDMS under optimized conditions. Subsequent reduction with DIBAL-H provides **60** in 91% yield. This route comprises four steps with an overall yield of 42%, representing the shortest sequence and the highest yield among all reported routes. However, the high cost of the substrate **57**, as well as the expenses associated with cryogenic reactions and fluorinating reagents, may need to be evaluated for industrial production. This electrophilic fluorination approach at the 2-position of the furanose ring is concise and direct. Similar reports of two-step halogenations at the 2-position of furanose rings, including fluorochlorination and fluorobromination, have also been documented [26].

**Figure 5:**
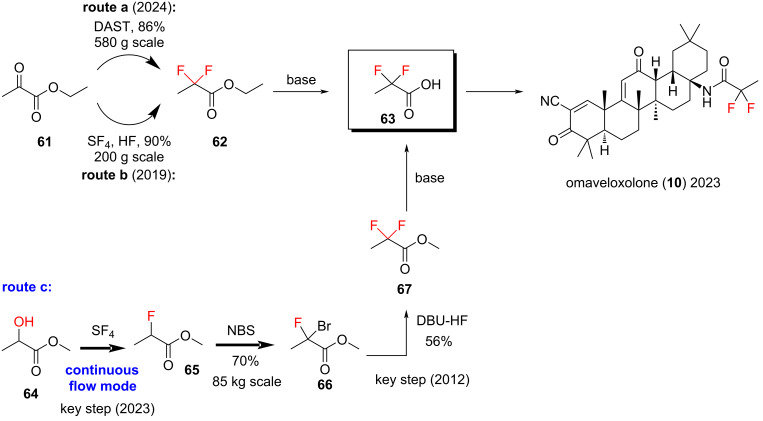
Synthesis of the difluorinated intermediate for omaveloxolone (**10**).

**1.1.4 Omaveloxolone (2023):** As shown in Figure 5, compound **63** is a key intermediate in the synthesis of omaveloxolone (**10**). 2,2-Difluoropropionic acid (**63**) can be produced at low cost. Two synthetic methods are described below, in which the transformation from **65** to **66** in route b has been scaled up to 85 kg.

Both route a (reported in 2024) and route b (reported in 2019) start from ethyl pyruvate (**61**) and use either DAST (590 g scale, 86% yield) [27] or SF_4_/HF (200 g scale, 90% yield) [28] for fluorination of the carbonyl group to give **62**, followed by hydrolysis to afford **63**. For the difluorination of β-keto esters, synthetic methods using SF_4_ have been reported [29], and safety and efficiency evaluations indicate that SF_4_ holds promise for such transformations. However, whether the continuous-flow difluorination of α-keto esters such as ethyl pyruvate (**61**) can be achieved remains to be investigated.

In route c, assembled from literature [17,30] reported between 2012 and 2023, the key CF_2_ introduction (reported in 2023) uses methyl 2-(*R*)-fluoropropanoate or the racemate **65** as the starting material. Compounds **65** can be obtained from methyl lactate (**64**) and SF_4_ in a continuous-flow reaction via a nucleophilic fluorination mechanism in 86% for the racemate and 81% for the *S*-enantiomer with 99% ee [17]). Under the action of a radical initiator, bromination is carried out with NBS (gas-phase purity after distillation 99.9%, 85 kg scale, yield 70%) to afford **66**. Subsequently, in the presence of a catalytic amount of 2,6-di-*tert*-butyl-4-methylphenol (BHT) and using 1,3-dimethyl-2-imidazolidinone (DMI) as the solvent, the bromine atom is substituted by fluorine using 1,8-diazabicyclo[5.4.0]undec-7-ene (DBU) and HF, generating **67** in 56% yield (223 g scale) [30]. The ratio of DBU to HF is a key process parameter in this step and the best results are achieved at a ratio of 1:2.6, whereas decreasing the HF equivalents leads to a significant increase in the by-product methyl 2-fluoroacrylate.

**Figure 6:**
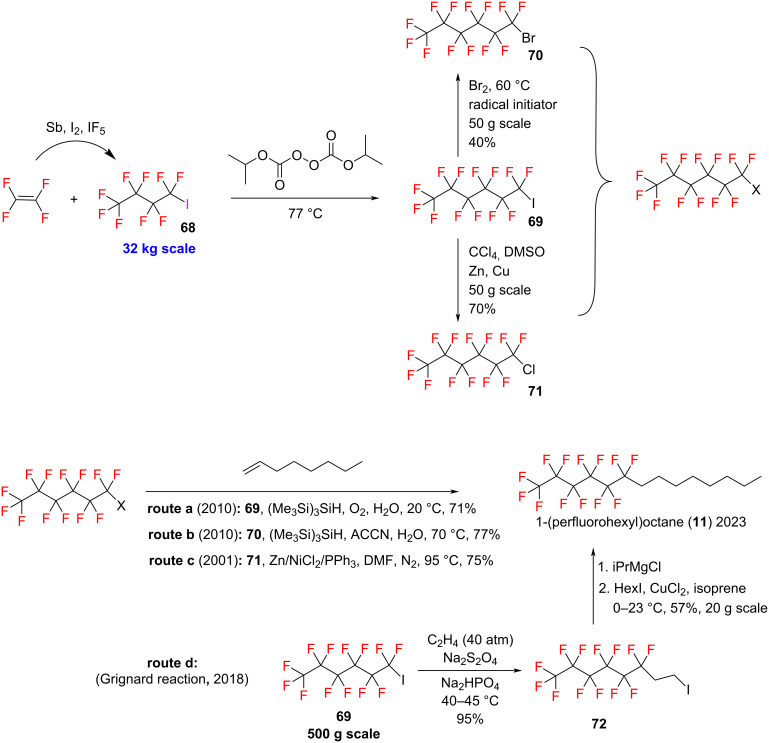
Synthesis of the difluorinated intermediate for 1-(perfluorohexyl)octane (**11**).

**1.1.5 1-(Perfluorohexyl)octane (2023):** As shown in Figure 6, C_6_F_13_X (X = Cl, Br, I) is the key CF_2_-containing fragment for the synthesis of 1-(perfluorohexyl)octane (**11**).

There are several methods for synthesizing C_6_F_13_I (**69**). For example, using C_4_F_9_I (**68**) and tetrafluoroethylene as starting materials, with diisopropyl peroxydicarbonate (or fluorine-containing peroxides such as (C_2_F_5_COO)_2_ [31]) as a radical initiator and C_2_F_5_I as a telogen, compound **69** can be obtained via telomerization on a 32 kg scale in approximately 30% yield [32]. Substrate **68** itself can be prepared from tetrafluoroethylene using IF_5_ and iodine under antimony catalysis at room temperature and 0.4 MPa, giving a yield of 97.2% and a purity of 99.6% [33].

Using **69** as the starting material, there are four routes for the synthesis of **11**: either **69** can be used directly, or first converted to the corresponding bromide or chloride, followed by radical addition with 1-octene to give the target product (routes a–c), or via a Grignard reaction (route d).

In route a (reported in 2010) [34], compound **69** is reacted with 1-octene in a single step to afford **11**. This reaction, proceeding via a radical-mediated perfluoroalkylation mechanism, is initiated by (Me_3_Si)_3_SiH and dioxygen to generate the C_6_F_13_**^·^** radical, which then adds to 1-octene and abstracts a hydrogen atom from silane to afford the target product **11** (5 × 10^−5^ mol scale). This method uses no organic solvent, is environmentally friendly and gives a yield of 71%; however, the experimental scale is small and the cost of (Me_3_Si)_3_SiH is relatively high.

In route b (reported in 2010) [34], using C_6_F_13_Br (**70**) as the starting material under the same conditions as route a gives compound **11** in only 21% yield. However, when 1,1'-azobis(cyclohexane-1-carbonitrile) (ACCN) is used as the radical initiator instead of oxygen, the yield can be increased to 77% at 70 °C. The starting compound **70** can be synthesized from **69** by bromination in 40% yield [35].

Route c (reported in 2001) uses C_6_F_13_Cl (**71**) as the starting material. Under reduction by Ni(PPh_3_)_4_ (prepared from Zn powder, NiCl_2_, and PPh_3_), a single-electron transfer (SET) generates the C_6_F_13_**^·^** radical, which then adds to 1-octene to afford **11** in 75% yield [36]. Substrate **71** can be synthesized from **69** by reaction with CCl_4_ in the presence of a Zn–Cu couple [37], with 70% yield [38].

In addition to the above three radical routes, route d is based on a Grignard reaction (reported in 2018). Here, compound **72** (synthesized from **69**, 95% yield [39]) undergoes a Grignard exchange reaction with isopropylmagnesium iodide to form the corresponding Grignard reagent, which is further reacted with iodohexane in the presence of a catalytic amount of CuCl_2_ to extend the carbon chain, affording 1-(perfluorohexyl)octane (**11**) in 57% yield on a 20 g scale [40].

In summary, C_6_F_13_X (X = Cl, Br, I) all are suitable substrates reacting with 1-octene to produce 1-(perfluorohexyl)octane. However, the iodo-substituted compound **69** has lower cost and is more suitable for industrial production. Route d is based on a Grignard approach and involves high-pressure reaction conditions (40 atm ethylene). Route a, in contrast, proceeds via a radical-mediated perfluoroalkylation in water under mild conditions but uses (Me_3_Si)_3_SiH as a relatively expensive reagent and the reported reaction was conducted on a very small scale (5 × 10^−5^ mol).

**Figure 7:**
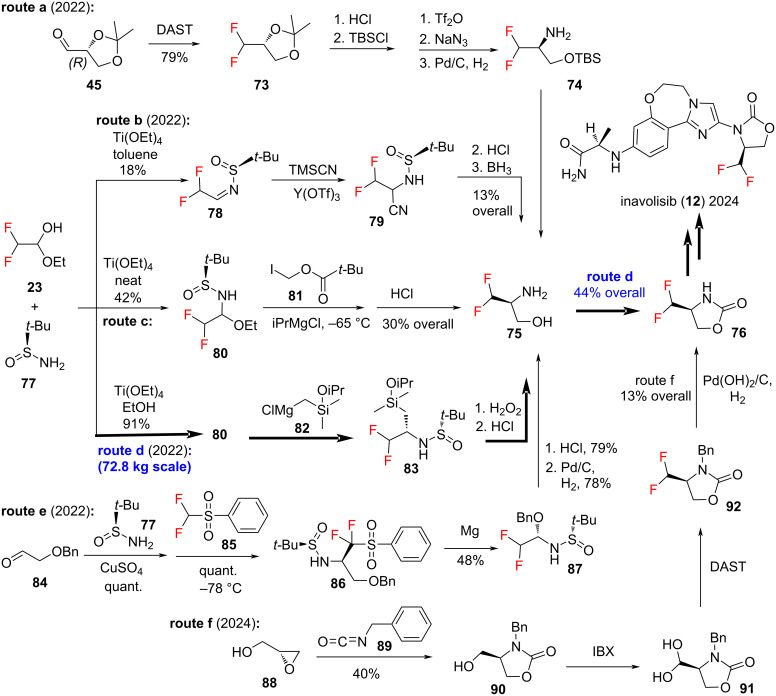
Synthetic routes towards difluorinated intermediates for the synthesis of inavolisib.

**1.1.6 Inavolisib (2024):** As shown in Figure 7, the chiral β-amino alcohol **75** containing a difluoromethyl group is a key intermediate in the synthesis of inavolisib (**12**).

Route a (reported in 2022) [41] starts from chiral aldehyde **45** and introduces the CF_2_ group via fluorination with DAST. This deoxyfluorination gave intermediate **73** in 79% yield, but route a suffers from several major issues: the use of hazardous reagents such as DAST, sodium azide, and phosgene, as well as low yields in the deacetalization and silyl protection of the primary alcohol steps.

Routes b–d (reported in 2022) [9] all employed building block **23** to introduce the CF_2_ group, with route d being the industrial route. Using **23** and *tert*-butylsulfinamide (**77**), the chiral nitrogen center was constructed under Ti(OEt)_4_ catalysis under different conditions: using toluene as solvent under reflux conditions afforded product **78** in 18% yield associated with significant decomposition to hydrogen fluoride (route b), reaction under neat conditions (route c) or using ethanol as solvent (route d) gave product **80** in 42% (solvent-free) or 91% yield (in ethanol), respectively. In route b, **78** underwent chiral cyano addition to the imine under 0.1 equiv of Y(OTf)_3_ catalysis, with stereoselectivity highly dependent on the Lewis acid catalyst, followed by hydrolysis and reduction to give the chiral amino alcohol (containing 11% enantiomer). In route c, intermediate **80 –** upon treatment with iodomethyl pivalate and isopropylmagnesium chloride – undergoes first elimination of ethanol to generate the imine in situ, followed by addition and hydrolysis of the *tert-*butyl ester, conveniently delivering **75** as a single stereoisomer in 30% yield. However, this step requires a low temperature of −65 °C, and the iodomethyl pivalate tends to form gel-like spheres under low-temperature magnetic stirring, which is detrimental to scale-up operations. Route d utilizes **82** and **80** to form **83** via a six-membered ring transition state, followed by Fleming–Tamao oxidation [42] to convert **83** into the chiral amino alcohol **75**. Compared with the other routes, route d features mild reaction conditions and has been scaled up to 72.8 kg with an overall yield of 44%.

Route e (reported in 2022) [9] uses (difluoromethylsulfonyl)benzene (**85**) as fluorinating reagent to introduce the difluoromethyl group. First, compound **84** formed an imine with (*S*)-*tert*-butylsulfinamide quantitatively in the presence of copper sulfate, which was then added to **85**, followed by reduction with magnesium turnings to introduce the CF_2_ group. The addition step was clean, with no minor isomer detected, but required −78 °C and the reduction step gave a low yield of only 48%.

Route f (reported in 2024) [43] requires only four steps to obtain **76**. The chiral starting material **88** reacted with isocyanate **89** to give **90** in one step. The primary alcohol was then oxidized with 2-iodoxybenzoic acid (IBX) to give **91**, which was then treated with DAST to introduce the CF_2_ group, affording **92**. Final debenzylation gave **76** in approximately 13% overall yield on a gram scale.

In summary, compared with other routes, route d (building block approach for introducing CF_2_) offers significant advantages. This route uses no expensive or hazardous reagents such as DAST, exhibits excellent chiral selectivity and high yield, and it has been successfully scaled up to 72.8 kg. It provides a practical and feasible reference for the synthesis of chiral β-aminoalcohols containing CF_2_ groups such as **75**.

Beyond the alkyl–CF_2_ construction strategies employed in the six approved drugs discussed above, the *gem*-difluorocyclopropane motif has gained increasing attention as another emerging CF_2_-containing building block in medicinal chemistry. The *gem*-difluorocyclopropane ring combines the unique metabolic stability and conformational effects of the CF_2_ group with the inherent strain and structural rigidity of a cyclopropane, offering a distinct profile for modulating drug-like properties. Synthetic access to these compounds was comprehensively summarized by Volochnyuk and Grygorenko in 2020 [44], and the [2 + 1] cycloaddition of difluorocarbene with alkenes remains the predominant method for preparing *gem*-difluorocyclopropanes. For instance, a kilogram-scale synthesis of the difluorocarbene precursor Ph_3_P^+^CF_2_CO_2_^−^ was reported in 2024 [45], and a large-scale cyclopropanation of butyl acrylate with difluorocarbene, combined with classical resolution of the resulting fluorinated building block, was demonstrated in 2022 [46]. The landscape of commonly used reagents is evolving, with novel protocols based on trimethylsilyl fluorosulfonyldifluoroacetate (TFDA), the Ruppert–Prakash reagent (CF_3_SiMe_3_), and BrCF_2_SiMe_3_ gaining momentum, particularly with their extension to functionalized substrates. More recently, the facile synthesis of chiral *gem*-difluorocyclopropanes via rhodium-catalyzed hydrogenation was reported, highlighting the potential for enantioselective construction of these motifs [47]. Although none of the 12 FDA-approved drugs discussed in this review currently feature a *gem*-difluorocyclopropane, this motif represents a promising frontier in CF_2_-containing drug discovery, and the demonstrated process-scale methodologies suggest its potential viability in future industrial route evaluations.

#### 1.2 Construction strategies for heteroaryl–CF_2_

Direct CF_2_ functionalization of aromatic rings typically employs metal-catalyzed methods, such as the reaction of ethyl bromodifluoroacetate with 2-haloarenes in the presence of copper powder, or metal-catalyzed coupling using difluoromethyl 2-pyridylsulfone (Hu's Reagent). Among the four drugs discussed in this section, oteseconazole employs the aforementioned metal-catalyzed strategy, while the CF_2_ groups in glecaprevir and voxilaprevir, both attached to a pyrazine ring, are introduced via a building block approach, whereas the CF_2_ group in the structure of lenacapavir attached to a pyrazole ring is introduced via indirect fluorination of a carbonyl group.

**Figure 8:**
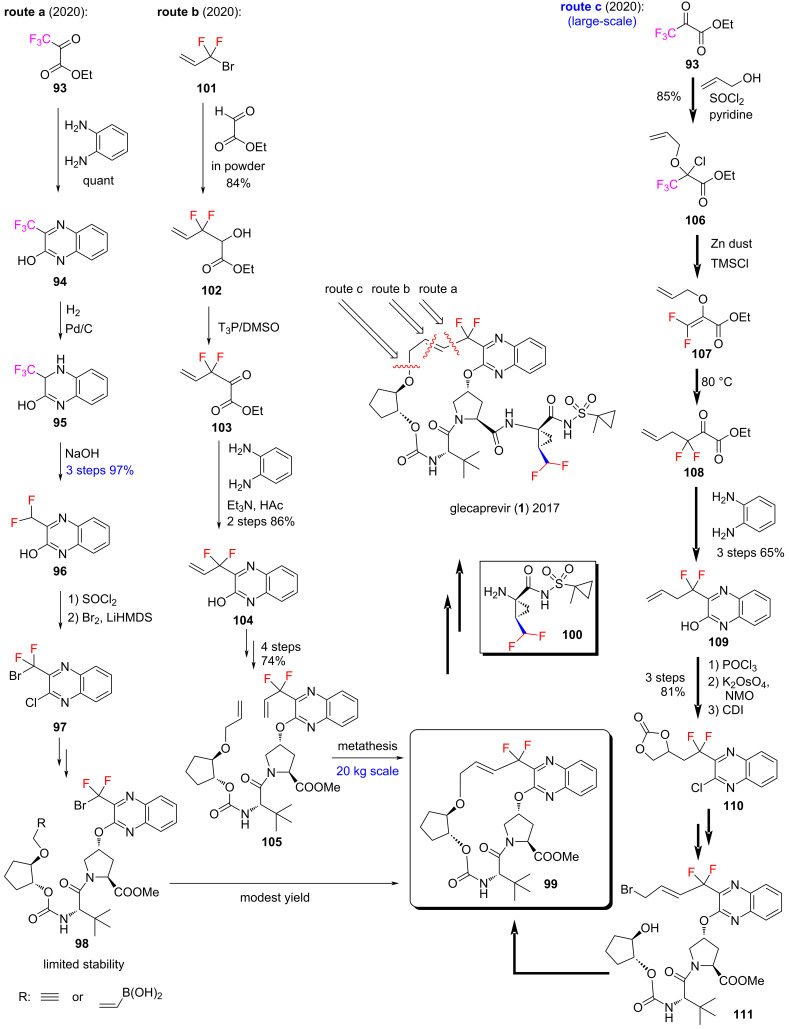
Synthesis of the difluorinated intermediate for glecaprevir.

**Figure 9:**
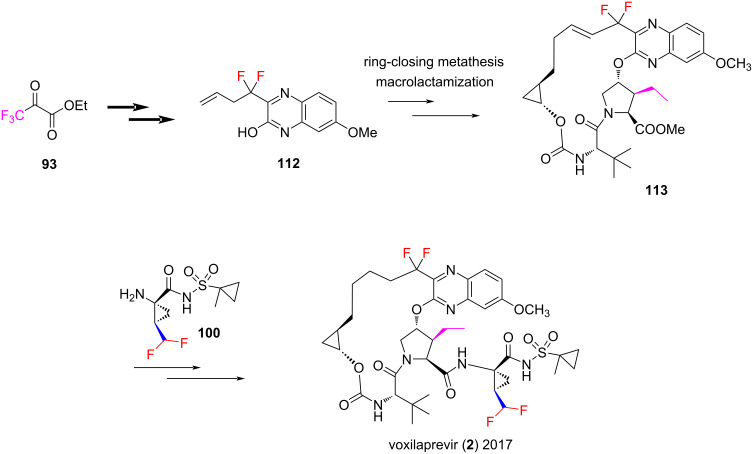
Synthesis of the difluorinated intermediate for voxilaprevir.

**1.2.1 Glecaprevir (synthesis of the heteroaryl–CF_2_ main chain):** As shown in Figure 8, route a constructs the heteroaryl–CF_2_ moiety via HF elimination from a CF_3_-containing heteroaromatic ring, while route b directly introduces the CF_2_ group using a difluorinated building block. Both routes, however, suffer from significant process drawbacks. Route c starts from the same building block as route a but employs a fundamentally different dehalogenation strategy and was ultimately selected as the large-scale manufacturing route. The analogous synthetic route for voxilaprevir is presented in Figure 9 for direct comparison (see section 1.2.2 for discussion).

Route a (reported in 2020) [48] adopts an HF elimination strategy: the CF_3_-containing starting material **93** undergoes quantitative ring closure with *o*-phenylenediamine, followed by hydrogenation and HF elimination with sodium hydroxide, affording the aryl–CF_2_ intermediate **96** in 97% yield over three steps. However, when three strategies – a Heck reaction, Sonogashira-type reaction, and Suzuki reaction – were subsequently explored for macrocyclization, the allylic difluoride moiety in product **99** was found to be highly prone to HF elimination under palladium-catalyzed coupling conditions. The best yield achieved by this method was only 62%, while the worst was as low as 11%. Further studies revealed that even the purified coupling product **99** decomposed by 67% within just 1 hour under the optimal reaction conditions (0.015 equiv Pd_2_(dba)_3_, 0.07 equiv Ruphos, 60 °C), indicating that this structure is inherently unstable under such coupling conditions.

Route b (reported in 2020) [6] uses a CF_2_-containing building block **101** to generate the α,α-difluoroallyl carbanion in the presence of powdered indium, followed by condensation with ethyl glyoxylate to give **102** [49]. Subsequent oxidation and ring closure afford the aryl–CF_2_ intermediate **104** which was then converted to **105** in four steps with 74% yield. Next, intermediate **105** underwent ring-closing metathesis (RCM) to form the macrocycle **99**, followed by coupling with **100** (synthesized from **18**) to afford compound **1** (41 kg). However, this route required multiple silica gel purifications and recrystallizations, and the two-step yield of the RCM and hydrolysis was only 60%, limiting further scale-up. Points to note for the CF_2_-introduction step: indium powder tends to settle at the bottom and is oxygen-sensitive, thus requiring proper agitator design to ensure mixing and degassing of the solvent.

Route c (reported in 2020) employs an intramolecular etherification macrocyclization strategy to achieve large-scale industrial production [48]. Still starting from **93**, the strongly electron-deficient ketone carbonyl undergoes addition with allyl alcohol, followed by chlorination with SOCl_2_ to give **106** (85% yield over two steps). Subsequent reduction with activated zinc powder and Claisen rearrangement afford the key fragment, δ,ε-unsaturated β,β-difluoro-α-keto ester **108** (this method was reported in 1995 [50], zinc powder reduction is superior to base elimination). Ring closure with *o*-phenylenediamine gives **109** (65% yield over three steps). Subsequent dihydroxylation, cyclic carbonate formation, and further transformations afford **111**, followed by intramolecular etherification to close the macrocycle, yielding **99**. Key scale-up process points for the CF_2_ group introduction are: 1) pyridine should be added beforehand to the addition reaction to prevent HF generation (from trace fluorohydrin impurity in the starting material reacting with allyl alcohol), which would corrode equipment; 2) during activation of zinc powder with TMSCl, oxygen content must be strictly controlled below 50 ppm; 3) the activated zinc slurry must be added portionwise to avoid excessive heating and formation of overreduction impurities. Additionally, the slurry can be completely transferred through modification of the reactor design.

In summary, for the building block approach, the choice of fluorine-containing building block must consider both the construction of the aryl–CF_2_ moiety and the overall molecular assembly strategy. Regarding the construction of the aryl–CF_2_ moiety alone, the methods from all three routes are worthy of reference. Furthermore, the HF elimination approach in route a has limited applicability. When CF_3_ is directly attached to an aromatic ring such as a benzene or 3-pyridine ring, the fluoride-initiated coupling reaction between trifluoromethylarenes and allylsilanes [51] may represent an alternative approach for preparing allylated α,α-difluorobenzyl compounds like **109**. A 2022 review provides a broader methodological overview of constructing CF_2_ architectures from CF_3_ groups [52].

**1.2.2 Voxilaprevir (2017):** As shown in Figure 9, the chemical environment of the CF_2_ group in voxilaprevir (**2**) is highly similar to that in glecaprevir (**1**), and the synthetic route is essentially the same. The same rearrangement-based strategy (reported in 2017) for constructing the aryl–CF_2_ moiety as described in route c for glecaprevir was adopted [53], and will not be repeated here.

**Figure 10:**
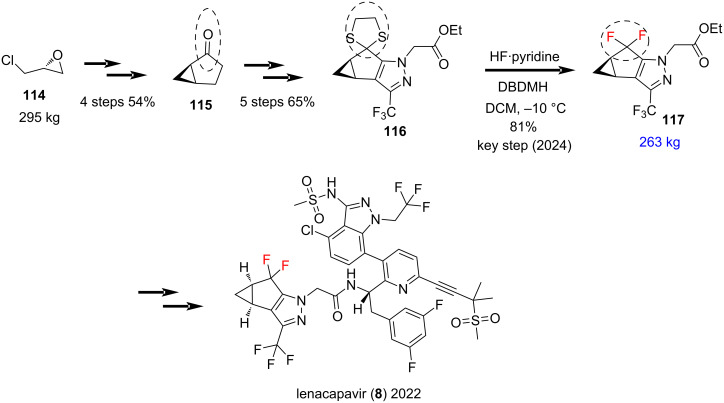
Synthesis of the difluorinated intermediate for lenacapavir.

**1.2.3 Lenacapavir (2022):** The pyrazole ring in the lenacapavir (**8**) molecule is constructed via a cyclization reaction, and the CF_2_ group on the ring originates from the ketone carbonyl of compound **115** (synthesized from **114** in 4 steps, 54% yield, on a scale of 295 kg [54]) (Figure 10). Clearly, the DAST fluorination route is unsuitable for industrial requirements.

A literature report [55] described an indirect difluorination method for ketones: first converting the ketone to a dithioketal, then oxidizing it with 1,3-dibromo-5,5-dimethylhydantoin (DBDMH), followed by fluorination with 70 wt % HF·pyridine (Olah's reagent). Nearly four decades later, this classic method was successfully applied to the synthesis of the key intermediate **117** of lenacapavir (**8**) on a scale of approximately 300 kg, with both the dithioketal protection and fluorination steps achieving yields greater than 80% (reported in 2024) [54]. The fluorination proceeds via a nucleophilic fluorination mechanism. The authors systematically screened both oxidants and fluorinating reagents in the fluorination step. Among the oxidants, *N*-bromosuccinimide (NBS) and others were evaluated, but DBDMH was chosen as the best option for multi-kilogram production due to its low cost, stable supply, and double the oxidizing equivalents. Among fluorinating reagents, 70 wt % HF·pyridine, triethylamine hydrofluoride, *N*,*N*'-dimethylpropyleneurea (DMPU)·HF, *N*,*N*-diethyl-1,1,2,3,3,3-hexafluoropropylamine (Ishikawa's reagent), and alkali metal fluorides were compared, and Olah's reagent was ultimately determined to give the best results. The optimized conditions employed 3.4 equivalents of DBDMH and 17 equivalents of 70% HF·pyridine, affording the target product with high purity at the reaction endpoint and an acceptable yield (81%).

In the CF_2_-introduction reaction for lenacapavir (**8**), several key process points merit attention and provide general guidance for the construction of CF_2_ groups in similar structures: 1) The quenching step in the fluorination process is highly exothermic; adequate cooling measures must be implemented (in the literature, a Mettler Toledo EasySampler was used for real-time monitoring) to avoid impurity formation. 2) The reaction system must be thoroughly dried and free of methanol. Otherwise, the activated intermediate will hydrolyze back to the ketone, leading to fluorination failure (a commercial batch-experienced reduced yield for this reason). 3) DBDMH contains trace amounts of chlorine, which can introduce chlorine-containing impurities and must be controlled.

**Figure 11:**
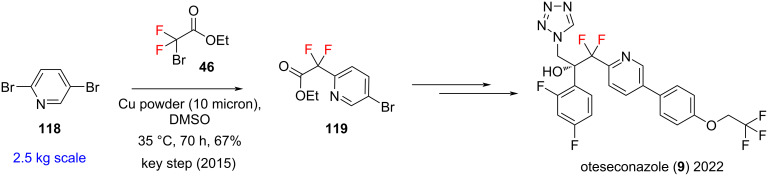
Synthesis of the difluorinated intermediate for oteseconazole.

**1.2.4 Oteseconazole (2022):** Ashwood et al. first reported the introduction of a difluoromethylene group at the 2-position of pyridine using ethyl bromodifluoroacetate and 2-halopyridine in the presence of copper powder [56]. As shown in Figure 11, the synthesis of the key intermediate **119** of oteseconazole (**9**) employs precisely this method. The reaction proceeds via a radical-mediated mechanism.

The patent [57] systematically investigated process parameters including copper-powder particle size and equivalents, solvent type, reaction temperature, and time. In a 2.5 kg scale-up experiment (reported in 2015) [58], the equivalents of ethyl bromodifluoroacetate (**46**) and copper powder were both reduced by half (for complete reaction 2.1 equivalents of copper were required, one equivalent giving only a 50% conversion of 2-bromopyridine to the pyridyldifluoroacetate [56]) compared to the early route [59], achieving a yield of 67%. One of the by-products was the disubstituted product in which both bromine atoms were replaced. A study has assessed the exothermic safety hazards of this type of reaction [60], noting that cooling measures or replacement with a more stable solvent are required. However, that report did not include data for 2,5-dibromopyridine (**118**); the substrate most similar to compound **118** investigated was 2-bromo-5-chloropyridine.

Hu's reagent (2-PySO_2_CF_2_H) has been a prominent research focus in recent years for the introduction of difluoromethylene groups onto aromatic rings. The direct construction of aryl–CF_2_ bonds from aryl bromides remains a more challenging transformation. A recent study reported a nickel-catalyzed cross-electrophile coupling reaction between (hetero)aryl bromides and Hu's reagent [61]. This reaction proceeds via a radical-mediated cross-electrophile coupling mechanism and operates under mild conditions (40 °C) and is suitable for small-scale parallel synthesis and bench-top scale-up. The study covered 24 (hetero)aromatic substrates, including benzene and pyridine rings, with an average yield of 67 ± 16%. Among them, the substrate most structurally similar to **118** was 2-bromo-3-methoxypyridine, which gave the corresponding heteroaryl–CF_2_ product in 62% yield. Whether this method can be applied to the synthesis of oteseconazole (**9**) remains to be further investigated. With scale-up in mind, the authors also optimized the synthesis of Hu's reagent: reaction of 2-chloro-2,2-difluoroacetophenone with pyridine-2(1*H*)-thione first generated a thioether, which was then oxidized to the sulfone using NaIO_4_ and RuCl_3_. This process required no chromatographic purification, and after crystallization, the two-step yield was 73% on a 1.4 g scale. Although the differential scanning calorimetry (DSC) onset and energy places 2-PySO_2_CF_2_H above the Pfizer modified Yoshida thresholds, the compound structure does not contain a high energy functional or explosive group. Therefore, the compound is considered not shock sensitive and not explosive. It should be noted that this reaction system requires 0.11 equivalents of the ligand pyridine-2,6-bis(carboximidamide) dihydrochloride (PyBCam), which is relatively expensive. In addition, the reaction requires 8 equivalents of zinc powder (the authors themselves acknowledged the limitations of zinc powder upon scale-up and recommended electrochemical reduction as an alternative [62]).

#### 1.3 Construction of ArO–CF_2_

Various methods have been reported for the construction of ArO–CF_2_ bonds from phenolic hydroxy groups. Examples include the use of difluorocarbene reagents such as TMSCF_2_X (X = Br, F, Cl) [63], or the formation of a thiocarbonate using CSCl_2_ followed by conversion to OCF_2_Cl with BrF_3_ and subsequent reduction with tributyltin hydride to afford the PhO–CF_2_ structure [64]. However, these methods involve reagents that are either relatively expensive (TMSCF_2_X) or highly hazardous (BrF_3_), which may limit their practicality at production scale. Currently, the more commonly reported strategy in industry is the use of ClCF_2_COONa as a difluorocarbene precursor. Furthermore, for catechol-type substrates, the construction of the CF_2_ group in difluorobenzodioxole rings using CSCl_2_ and AgF proceeds under relatively mild conditions.

**Figure 12:**
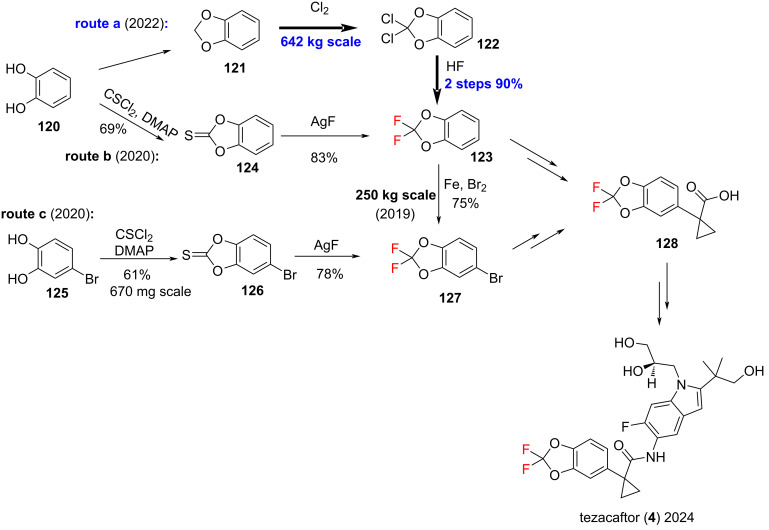
Synthesis of the difluorinated intermediate for tezacaftor.

**1.3.1 Tezacaftor (2018):** As shown in Figure 12, there are two methods for constructing the CF_2_ group in the key intermediates **123** and **127** of tezacaftor (**4**): the HF fluorination method (route a) and the AgF fluorination method (routes b and c).

Route a (reported in 2022) first synthesizes inexpensive 1,3-benzodioxole (**121**), then subjects the methylene group to dichlorination followed by difluorination. On a 642 kg scale, the two-step yield for dichlorination and difluorination was 90% [65]. However, the fluorination step uses hazardous reagents such as hydrogen fluoride. Route b (reported in 2020) first converts catechol (**120**) into thiocarbonyl ester **124** using CSCl_2_ (69% yield), then affords compound **123**, which contains a difluoromethylene group, via a difluorocarbene-mediated O–CF_2_ bond-formation mechanism involving a monofluorocarbenium ion intermediate generated with AgF (83% yield) [66]. The yield of this route is slightly lower than that of route a, but it avoids the use of HF and features milder reaction conditions and a shorter reaction time. It should be noted that silver fluoride requires activation, dehydration, and grinding under light-protected conditions. Route c (reported in 2020) starts from bromocatechol **125** and employs a strategy similar to route b, sequentially affording **126** (61% yield) and **127** (78% yield) [66]. In addition, **127** can be obtained from **123** via iron powder-catalyzed bromination with bromine; this transformation has been reported on a 250 kg scale with 75% yield [67].

Overall, route a has been scaled up to 642 kg. Although it involves hazardous reagents such as chlorine gas and HF, these are mature operations in the chemical industry and the risks are controllable. Routes b and c use the CSCl_2_/AgF method to construct the CF_2_ group in the difluorobenzodioxole ring, which is relatively mild. However, the special pretreatment required for silver fluoride needs comprehensive evaluation for industrial production.

**Figure 13:**
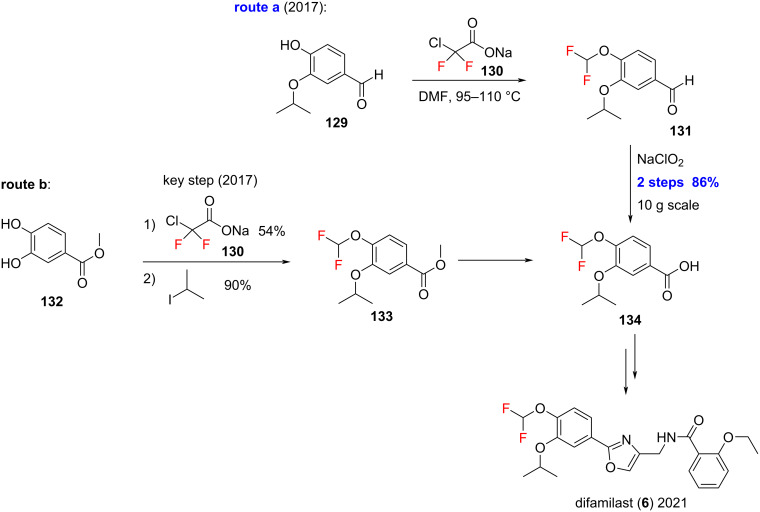
Synthesis of the difluorinated intermediate for difamilas (**6**).

**1.3.2 Difamilast (2021):** As shown in Figure 13, the synthesis of the key intermediate of difamilast (**6**) mainly involves the assembly of three functional groups: the difluoromethyl group, the isopropyl ether, and the carboxylic acid group.

Route a (reported in 2017) [68] uses a DMF solution of ClCF_2_COONa (**130**) as a difluorocarbene precursor to perform the difluoromethylation of the phenolic hydroxy group of compound **129** under basic, high-temperature conditions. This step proceeds via a difluorocarbene-mediated O–CF_2_ bond-formation mechanism, affording **131**. Subsequent oxidation of the aldehyde group to carboxylic acid **134** via the Pinnick oxidation gave a two-step yield of 86% on a 10 g scale. Route b selectively difluoromethylates the phenolic hydroxy group of **132** using a method similar to route a, achieving a yield of 54% (reported in 2017) [69], followed by isopropyl etherification to obtain **133** in 90% yield (reported in 2023) [70].

Furthermore, for the substrate methyl 4-hydroxy-3-iodobenzoate, a difluoromethylation method using ClCF_2_COONa similar to that in route a has been reported on a 7 kg scale [71]. This process allows precise control of exothermicity and CO_2_ release, giving a 99% yield. Systematic safety assessments and product stability studies (which showed that degradation impurities such as dimeric by-products do not increase significantly under the reaction conditions) have been conducted, indirectly confirming the feasibility of scaling up this type of reaction. However, a route starting from this substrate via phenolic difluoromethylation followed by isopropyl etherification to prepare **133** has not yet been reported.

**1.3.3 Asciminib (2021):** The CF_2_X group, serving as a non-conventional halogen-bond donor, plays an important role in medicinal chemistry, chemical biology, and drug discovery, and its synthetic methods have attracted increasing attention [72]. Traditional methods for synthesizing chlorodifluoromethyl aryl ethers include nucleophilic fluorination of trichloromethyl aryl ethers or aryl chlorothioformates, as well as photochlorination of difluoromethyl aryl ethers [73]. However, these methods generally suffer from issues such as high reagent hazard and harsh reaction conditions.

**Figure 14:**
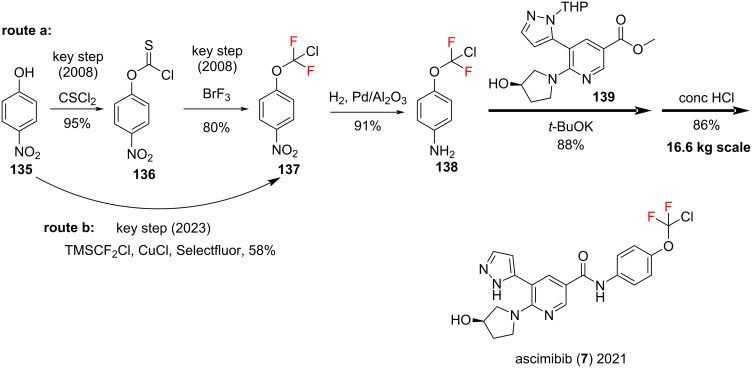
Synthesis of the difluorinated intermediate for asciminib.

As shown in Figure 14, route a, assembled from literature [74–76], was reported between 2002 and 2025, with the key CF_2_ introduction reported in 2008.

Route a first forms ester **136** from CSCl_2_ and *p*-nitrophenol (**135**) in 95% yield, followed by nucleophilic fluorination with BrF_3_ to afford the CF_2_Cl-containing intermediate **137** (80% yield, mmol scale) [74]. Subsequently, hydrogenation to reduce the nitro group (91% yield) [75] gives the key intermediate **138**. Compound **138** is then subjected to aminolysis of the ester in **139** (88% yield) and removal of the tetrahydropyranyl (THP) protecting group (86% yield) to afford 16.6 kg of asciminib (**7**) [76]. In this route, the fourth step applied 9.88 kg of compound **138** as the input, indicating that this intermediate has been produced on an industrial scale. However, the specific synthetic route to **138** was not disclosed in this report. It should be noted that BrF_3_ used in route a is a strong oxidizing agent with high hazard, requiring thorough protective measures for large-scale production.

Selectfluor® (1-chloromethyl-4-fluoro-1,4-diazoniabicyclo[2.2.2]octane bis(tetrafluoroborate), also known as F-TEDA-BF_4_) is an electrophilic fluorinating reagent. Route b (key CF_2_ introduction reported in 2023) [73] employs oxidative construction of the ArO–CF_2_Cl or ArO–CF_2_Br bond from phenols using Selectfluor, with (CH_3_)_3_SiCF_2_X and CuX (X = Cl or Br) under mild reaction conditions. This transformation proceeds via a difluorocarbene-mediated O–CF_2_ bond-formation mechanism, converting **135** to **137** in a single step with 58% yield [73]. This method is also applicable to the CF_2_X functionalization of aliphatic alcohols [77]. However, the relatively high cost of TMSCF_2_Cl used in this approach requires further evaluation of its cost-effectiveness and scale-up feasibility.

### 2. Mechanistic discussion

#### 2.1 Fluorination mechanisms

Building on the synthetic routes surveyed in section 1, this section provides a more systematic overview of the reaction mechanisms that underpin the key CF_2_ bond-forming steps. Each mechanism is illustrated with specific examples from the drug syntheses discussed above, allowing the reader to connect the practical route selection with the underlying chemical principles. The mechanistic illustrations presented herein are all summarized and redrawn based on reported literature data.

**2.1.1 Nucleophilic fluorination mechanism:** Nucleophilic fluorination is one of the most widely applied fluorination methods among the 12 drugs covered in this review. It introduces the CF_2_ group primarily through deoxofluorination of carbonyl groups using fluorinating reagents. The fluorinating reagents employed include DAST, SF_4_, Olah's reagent, and DBU–HF. Among these, SF_4_ and Olah's reagent have been successfully applied in industrial-scale production, whereas DBU–HF has been demonstrated only at laboratory scale (223 g).

**Figure 15:**
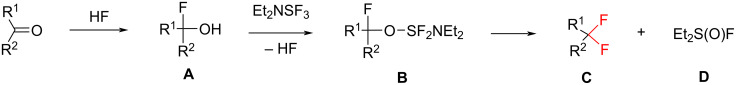
Mechanism of the deoxyfluorination of carbonyl groups using DAST.

The mechanism for the fluorination of carbonyl groups by DAST is illustrated in Figure 15 [78]. It is generally accepted that a small amount of HF present in the system first adds to the carbonyl group to generate intermediate **A**. Subsequently, the oxygen atom of the carbonyl group in **A** interacts with the sulfur atom of DAST to form intermediate **B**. The fluorine atom on the sulfur atom in **B** then attacks the carbon atom, expelling one molecule of **D** and simultaneously forming the difluorinated product **C**.

**Figure 16:**
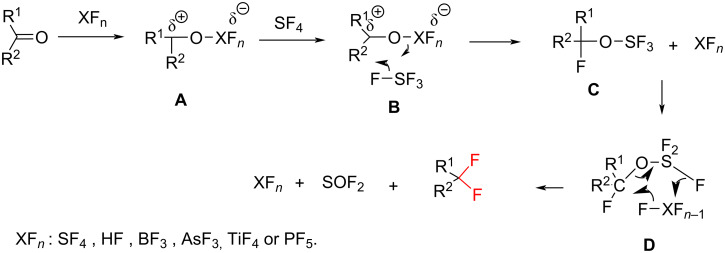
Mechanism of the deoxyfluorination of carbonyl groups using SF_4_.

The synthesis of compounds **37** (see section 1.1.2, route e in Figure 3), **62**, and **65** (see section 1.1.4 route b and c in Figure 5) employed SF_4_. The mechanism of deoxofluorination of carbonyl groups by SF_4_ is illustrated in Figure 16 [79]. The first step of the reaction involves the coordination of a fluoride source (XF*_n_*) with the carbonyl compound, polarizing the carbonyl group and forming intermediate **A**. SF_4_ itself can act as the fluoride source (XF*_n_*) in the reaction. Notably, more in-depth studies have revealed that the interaction between SF_4_ and the carbonyl group essentially proceeds through chalcogen bonding [80]. When a catalyst (such as HF, BF_3_, AsF_3_, TiF_4_, or PF_5_) is introduced into the system, the carbonyl group can be significantly activated via the formation of active species such as SF_3_^+^, allowing the reaction to proceed under milder conditions [81]. The effect of the catalyst is closely related to its Lewis acid strength toward the carbonyl group.

Subsequently, intermediate **A** then undergoes electron transfer with another molecule of SF_4_ via a cyclic intermediate **B** to form the monofluorinated intermediate **C**, which subsequently goes through a cyclic intermediate **D** to finally yield the difluorinated product. When R^1^ and R^2^ of the ketone are strong electron-withdrawing groups, the Lewis basicity of the carbonyl group is greatly weakened, leading to a significant decrease in coordination with the Lewis acid. Consequently, the fluorination reaction becomes more challenging and requires harsher conditions, such as elevated temperatures.

**Figure 17:**
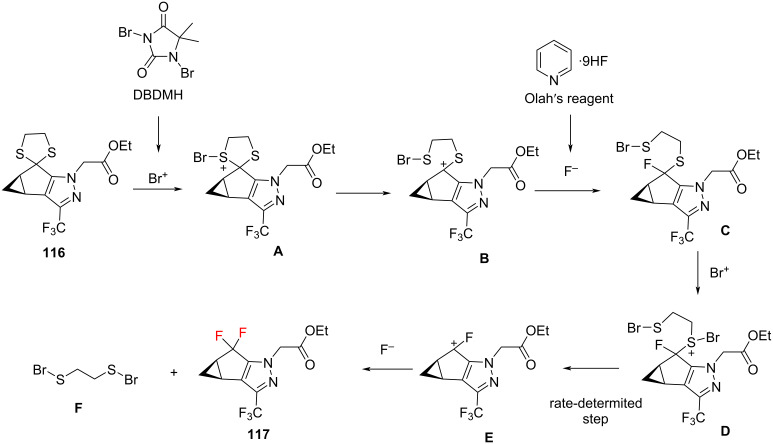
Mechanism of the fluorination of dithioketals using Olah’s reagent.

In the synthesis of compound **117** (see section 1.2.3, Figure 10), Olah's reagent was employed. The mechanism for the fluorination of dithioacetals by Olah's reagent is illustrated in Figure 17 [55]. The reaction is initiated by the attack of Br^+^, released from dibromodimethylhydantoin (DBDMH), on the sulfur atom of dithioacetal **116**, generating the bromosulfonium intermediate **A**. Intermediate **A** undergoes ring-opening to form the sulfur-stabilized carbocation **B**, which is subsequently trapped by a fluoride ion from Olah's reagent to yield the monofluorinated product **C**. Subsequently, a second equivalent of Br^+^ attacks the sulfur atom of intermediate **C**, forming the bromosulfonium ion **D** once again. Intermediate **D** then undergoes cleavage with desulfurization to generate the fluorine-stabilized carbocation **E**, which traps another fluoride ion to produce the difluorinated product **117**. The by-product of the reaction is 1,2-bis(sulfenyl bromide)ethane (**F**) or other species at an equivalent oxidation state. Because the electronegativity of the fluorine atom is significantly greater than that of sulfur, the fluorine-substituted carbocation **E** is less stable than the sulfur-substituted carbocation **B**; it is therefore hypothesized that the formation of **E** is the rate-determining step of the entire reaction sequence.

In the fluorination of compound **66** (see section 1.1.4, Figure 5), DBU-HF serves as a fluoride ion source, performing a nucleophilic substitution on the brominated carbon atom to form the difluoromethylene structure.

**Figure 18:**
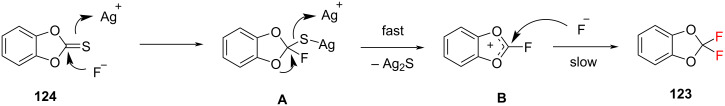
Mechanism of the fluorination of xanthates using AgF.

The synthesis of compound **123** (see section 1.3.1, Figure 12) employs silver fluoride as the fluorine source, and the mechanism is illustrated in Figure 18 [66]. AgF first undergoes addition with the C=S bond to generate intermediate **A**, which then rapidly extrudes Ag_2_S to form the oxocarbenium intermediate **B**. Intermediate **B** is subsequently nucleophilically attacked by a fluoride ion – this step being the rate-determining step of the overall reaction – to ultimately yield product **123** containing the CF_2_ moiety. Studies have indicated that the aforementioned oxocarbenium intermediates exhibit a certain degree of stability in solution.

**2.1.2 Electrophilic fluorination mechanism:** Among the 12 drugs discussed in this review, the electrophilic introduction of a difluoromethylene group is employed only in the synthesis of intermediate **59** of Cedazuridine (**5**) (see section 1.1.3, route f in Figure 4), with NFSI serving as the electrophilic fluorination reagent in this step. Umemoto et al. have provided a comprehensive historical review on the development of N–F fluorinating agents, including a discussion on the ongoing debate regarding the mechanism of NFSI [82]. Among the studies summarized, Timofeeva, Mayr, and co-worker reported kinetic evidence that, under metal-free conditions, the fluorination of carbanions with NFSI follows an S_N_2-type pathway rather than a SET process [83]. This S_N_2 pathway is therefore considered to be operative in the lactone enolate fluorination described herein.

**Figure 19:**
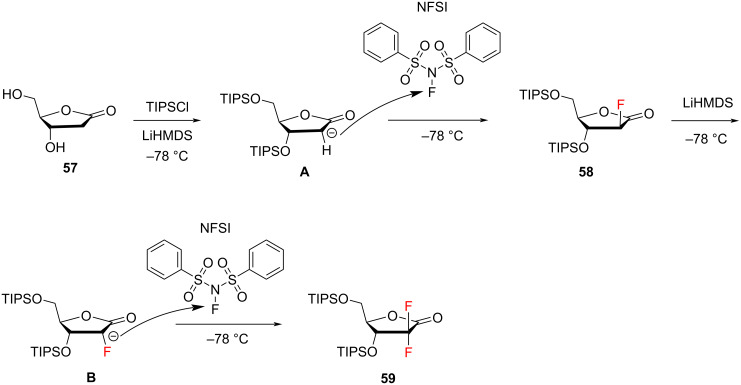
Mechanism of electrophilic fluorination using NFSI.

As shown in Figure 19, under the action of a strong base (LiHMDS) at −78 °C, deprotonation occurs at the C2 position of the sugar ring to generate the first carbanion species **A**. This intermediate then undergoes electrophilic fluorination with NFSI to afford the monofluorinated product. Subsequently, a second deprotonation with LiHMDS generates another carbanion **B** from the monofluorinated intermediate, which is again trapped by NFSI to form the difluorinated product **59**. In both steps, the carbanion acts as a nucleophile, attacking the electrophilic fluorine of NFSI to form the C–F bond.

It should be noted that this route remained only at the stage of laboratory exploration, the eventual industrial production adopted the Reformatsky reaction instead, introducing the CF_2_ group via the building block BrCF_2_COOEt and no longer involving an electrophilic fluorination pathway.

**2.1.3 Radical-mediated fluoroalkylation and cross-coupling mechanisms:***Copper-catalyzed system:* The synthesis of **119** from **118** employs a copper-catalyzed approach to construct the aryl–CF_2_ bond (see section 1.2.4, Figure 11). This reaction may involve a single-electron transfer process from copper powder to ethyl bromodifluoroacetate [84], which falls within the realm of radical mechanisms.

**Figure 20:**
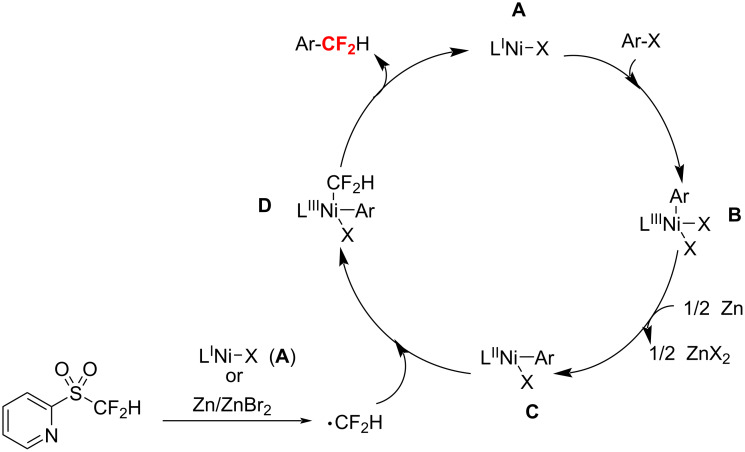
Mechanism of the Ni-catalyzed conversion of Ar–X to Ar–CF_2_H using Hu’s reagent.

*Nickel-catalyzed cross-electrophile coupling system:* The construction of the heteroaryl–CF_2_ bond from 2-bromo-3-methoxypyridine, a compound structurally similar to **118**, adopts a cross-electrophile coupling strategy using Hu's reagent under nickel catalysis. This reaction likewise proceeds via a radical-mediated cross-electrophile coupling mechanism, as illustrated in Figure 20 [61]: First, oxidative addition of the aryl halide ArX to the Ni(I) catalyst **A** generates a Ni(III) intermediate **B**, which undergoes reduction in the presence of Zn to afford a Ni(II) intermediate **C**. Concurrently, the difluoromethyl radical (^·^CF_2_H) is generated either by single-electron reduction of 2-PySO_2_CF_2_H by catalyst **A**, or by slow reduction mediated by Zn/ZnBr_2_ – this step being the rate-determining step of the entire catalytic cycle. Subsequently, ^·^CF_2_H undergoes oxidative addition to intermediate **C**, forming a Ni(III) intermediate **D**; **D** then undergoes reductive elimination to release the cross-coupling product ArCF_2_H, simultaneously regenerating catalyst **A** to complete the catalytic cycle.

**Figure 21:**
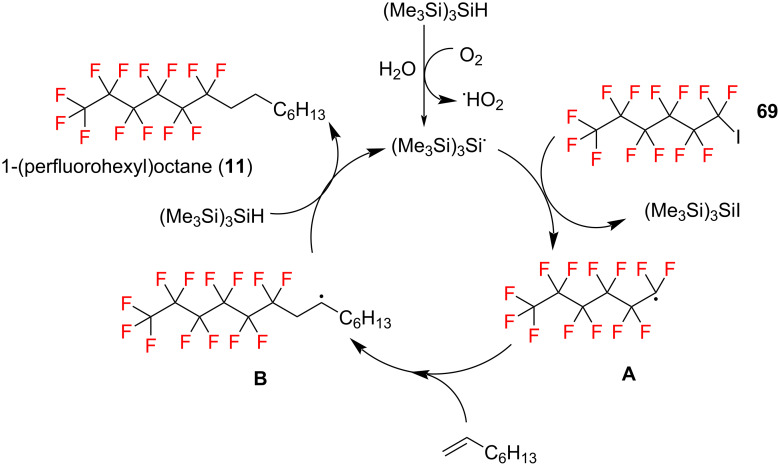
Mechanism of the (Me_3_Si)_3_SiH-mediated intermolecular perfluoroalkylation of alkenes in water.

The synthesis of **11** (see section 1.1.5, route a and b in Figure 6) involves a radical-mediated perfluoroalkylation mechanism. The mechanism of the (Me_3_Si)_3_SiH-mediated intermolecular perfluoroalkylation of alkenes in water is illustrated in Figure 21 [34]. The reaction is initiated by O_2_, which generates the (Me_3_Si)_3_Si^·^ radical, this radical abstracts an iodine atom from C_6_F_13_I (**69**) to generate the perfluoroalkyl radical **A**. The reaction of **A** with 1-octene proceeds faster than its reaction with the silane, preferentially forming the perfluoroalkylated radical adduct **B**. **B** subsequently abstracts a hydrogen atom from the silane to afford the target product, simultaneously regenerating the silyl radical to achieve chain propagation. For C_6_F_13_Br (**70**), the use of ACCN as the initiator in place of O_2_ provides superior results.

**Figure 22:**
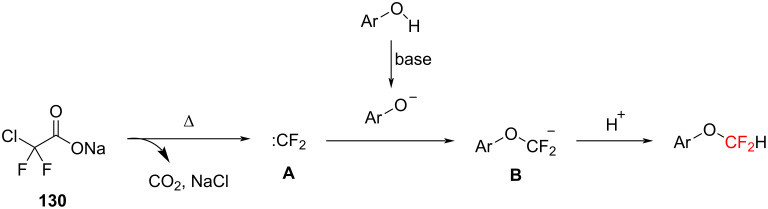
Mechanism of the formation of ArO–CF_2_H from ClF_2_COONa and ArOH.

**2.1.4 Difluorocarbene-mediated O–CF_2_ bond formation:** The syntheses of both **131** and **133** employ ClCF_2_COONa (**130**) as a difluorocarbene precursor (see section 1.3.2, Figure 13) and proceed via a difluorocarbene-mediated mechanism, as illustrated in Figure 22 [63,85]: Upon heating, **130** decomposes to generate CO_2_, NaCl, and difluorocarbene **A** (:CF_2_). Concurrently, the phenolic hydroxy group undergoes deprotonation under basic conditions to form the highly nucleophilic phenoxide, which subsequently attacks the electron-deficient difluorocarbene. The resulting intermediate **B** then undergoes protonation to ultimately afford the ArO–CF_2_H structure.

**Figure 23:**
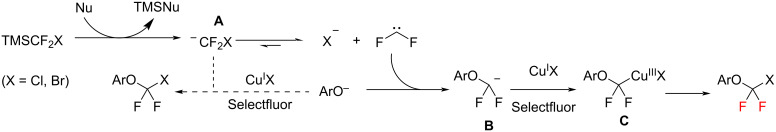
Mechanism of the formation of ArO–CF_2_X from TMSCF_2_X and ArOH.

The one-step synthesis of **137** from **135** (see section 1.3.3, route b in Figure 14) employs TMSCF_2_Cl and CuCl in the presence of a phenolic hydroxy group to construct the ArOCF_2_Cl structure, and also proceeds via a difluorocarbene-mediated mechanism, as illustrated in Figure 23 [73]: TMSCF_2_X (X = Cl, Br) first undergoes desilylation under the action of a nucleophile (phenoxide, halide, or BF_4_^−^) to generate the CF_2_X^−^ anion **A**, which subsequently undergoes α-elimination to produce difluorocarbene (:CF_2_) with concomitant release of a halide ion. The phenoxide then reacts with the difluorocarbene to afford the aryloxy–CF_2_ anion **B**. In the presence of the Selectfluor oxidant, **B** undergoes oxidative addition with CuX (X = Cl, Br), wherein Cu(I) is oxidized to Cu(III), forming a trivalent copper complex **C**. Finally, **C** undergoes reductive elimination to yield the target product, a chloro- or bromodifluoromethyl aryl ether, simultaneously regenerating the Cu(I) catalyst. Notably, an alternative pathway (indicated by dashed arrows in the Figure 23) – a copper-mediated direct oxidative coupling of the phenol with CF_2_X – is less favorable, because the CuCF_2_X complex is less stable than intermediate **C**, which disfavors the subsequent reductive elimination step.

In summary, difluorocarbene-mediated strategies have demonstrated practical utility in the construction of ArO–CF_2_ structures. For a broader perspective on difluorocarbene as a minimal perfluorocarbon linker in the synthesis of *gem*-difluoromethylenated compounds, readers are referred to a recent 2026 review [86].

As can be seen from the above analysis, nucleophilic introduction of the CF_2_ group is the most widely applied approach in industrial routes and exhibits the highest level of technological maturity. The method employing ClCF_2_COONa (**130**) as a carbene precursor for the construction of ArO–CF_2_ bonds from phenoxides has also found industrial application. In contrast, CF_2_-introduction reactions based on electrophilic and radical-mediated mechanisms are far less frequently adopted on an industrial scale.

#### 2.2 Summary of fluorination methods for CF_2_ groups in three different chemical environments

Key process steps and scale-up data of the discussed methods are summarized in Table 1.

**Table 1:** Summary of methods for constructing CF_2_ groups in FDA-approved drugs (2016–2025).^a^

Chemical environment	Example	Key raw material	Core method	Scale/yield	Remarks

alkyl–CF_2_	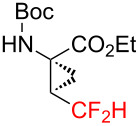 1.1.1 glecaprevir (**1**) Voxilaprevir (**2**) Figure 2, route d	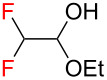	Knoevenagel reaction, Corey–Chaykovsky cyclopropanation	large scale/ 6 steps, 13%	DAST avoided
	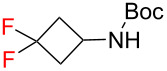 1.1.2 ivosidenib (**3**) Figure 3, route e	SF_4_ + Et_2_NH (continuous flow)	deoxyfluorination	gram scale/ 1 step, 68%	DAST made & used, safe
	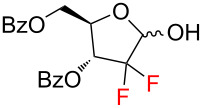 1.1.3 cedazuridine (**5**) Figure 4, route b	BrCF_2_COOEt	asymmetric Reformatsky reaction	commercial scale 6 steps, 21%	no hazardous reagent/ 25% chiral isomer
	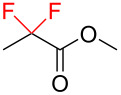 1.1.4 omaveloxolone (**10**) Figure 5, route c	SF_4_, NBS, DBU-HF	fluorination, bromination, substitution	85 kg 3 steps, 34%	good for scale-up
	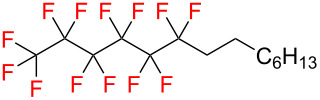 1.1.5 1-(perfluorohexyl)octane (**11**) Figure 6, route d	C_6_F_13_I	ethylene additon, Grignard coupling	20 g 2 steps, 54%	high pressure (40 atm ethylene)
	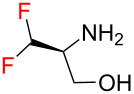 1.1.6 inavolisib (**12**) Figure 7, route d	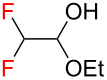	Ellman's sulfinamides, Tamao–Fleming oxidation	72.8 kg 4 steps, 44%	safe reagent, good stereoselectivity

heteroaryl–CF_2_	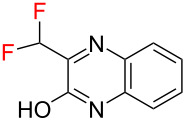 1.2.1 glecaprevir (**1**) Figure 8, route a	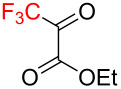	dearomatization, rearomatization (via HF elimination)	not reported 3 steps, 97%	downstream intermediates unstable
	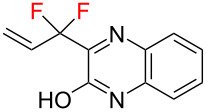 1.2.1 glecaprevir (**1**) Figure 8, route b	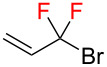	indium-mediation α,α-difluoroallyl carbanion	20 kg 3 steps, 72%	late-stage purification high cost
	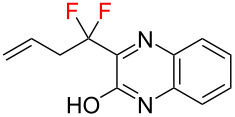 1.2.1 glecaprevir (**1**) Figure 8, route c	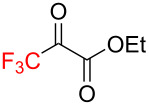	reductive dechlorofluorina- tion/Claisen rearrangement	large scale 4 steps, 55%	no hazardous fluorinating reagents, robust API late-stage assembly
	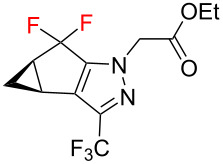 1.2.3 lenacapavir (**8**) Figure 10	DBDMH oxidation/ Olah’s reagent	dithioketal fluorination	263 kg, 81%	DAST avoided
	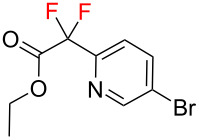 1.2.4 oteseconazole (**9**) Figure 11	BrCF_2_COOEt	copper-mediated cross-coupling	2.5 kg, 67%	exothermic safety risk

ArO–CF_2_	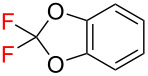 1.3.1 tezacaftor (**4**) Figure 12, route a, b	Cl_2_, HF	dichlorination difluorination	642 kg 2 steps, 91%	low cost/ uses hazardous reagent
		CSCl_2_, AgF	fluorodesulfuri- zation of thionobenzo- dioxoles with silver(I) fluoride	≈0.5 g 2 steps, ≈50%	mild conditions/ AgF pretreatment required
	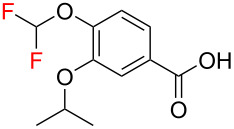 1.3.2 difamilast (**6**) Figure 13, route a, b	ClF_2_COONa	DMF-promoted difluorocarbene formation	11.8 g^b^ 2 steps, 86%	high yield, good safety
	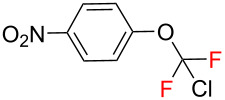 1.3.3 asciminib (**7**) Figure 14, route a, b	CSCl_2_, BrF_3_	nucleophilic fluorination	mmol scale 2 steps, 76%	high yield/ uses hazardous reagent
		TMSClF_2_, CuCl, Selectfluor	copper-mediated oxidative chlorodifluoro- methylation	≈100 mg 1 step, 58%	short steps, mild conditions/high cost

^a^For some drugs, no industrial route has been disclosed in the open literature; the routes listed in the table are provided for reference. ^b^This method has been reported at a kilogram scale, e.g. methyl 4-hydroxy-3-iodobenzoate.

## Conclusion

This review systematically surveyed the synthetic processes of 12 CF_2_-containing drugs approved by the FDA between 2016 and 2025, categorizing these drugs according to the chemical environment of CF_2_ (alkyl–CF_2_, heteroaryl–CF_2_, ArO–CF_2_), analyzing the selection logic behind each drug's industrial route and the main reasons why alternative routes were eliminated, and summarizing the four types of reaction mechanisms involved (nucleophilic fluorination, electrophilic fluorination, radical-mediated mechanism, and carbene mechanism). The main conclusions are as follows:

(1) Construction of alkyl–CF_2_: industrial routes exhibit a clear trend toward "avoiding direct carbonyl fluorination". High-risk reagents such as DAST have been gradually abandoned during scale-up due to safety concerns and low yields. Alternative approaches include: SF_4_/Et_2_NH continuous flow to generate and consume DAST in situ, direct SF_4_ continuous-flow fluorination, and the direct use of CF_2_-containing building blocks (e.g. compound **23**) to construct the target structure. Among these, the building block strategy has been preferentially adopted in the industrial routes for multiple drugs reviewed herein (e.g., glecaprevir, inavolisib) owing to its short step count and high safety profile.

(2) Construction of heteroaryl–CF_2_: metal-catalyzed direct introduction of the difluoromethylene group (e.g., Cu-mediated reaction of halopyridines with BrCF_2_COOEt, Ni-catalyzed cross-electrophile coupling with Hu's reagent) performs well on a laboratory scale, yet its industrial application remains constrained by ligand cost, exothermic risk, and purification challenges. Successfully industrialized cases (e.g., glecaprevir and lenacapavir) rely more heavily on building-block strategies or indirect fluorination methods (e.g., dearomatization–HF elimination of CF_3_ substrates, dithioketal fluorination, etc.).

(3) Construction of ArO–CF_2_: The route involving thermal decomposition of ClCF_2_COONa to generate difluorocarbene has been successfully scaled up (e.g., the difluoromethylation of methyl 4-hydroxy-3-iodobenzoate, a substrate analogous to that used in the synthesis of difamilast intermediate, has reached a 7 kg scale), featuring simple operation and low cost. The TMSCF_2_Cl/Cu-catalyzed oxidative coupling approach for synthesizing ArOCF_2_Cl proceeds under mild conditions and in short steps; however, the reagent cost remains relatively high and its feasibility for scale-up awaits further validation. For the construction of the difluorobenzodioxole ring in tezacaftor, the Cl_2_/HF fluorination method is mature and reliable, with a demonstrated scale of up to 642 kg, whereas the AgF-mediated fluorination method proceeds under milder conditions but gives a lower yield, requiring a comprehensive evaluation.

Overall trend: In the process development of the 12 marketed drugs reviewed, the industry shows a clear preference for preformed CF_2_-containing building blocks or indirect fluorination strategies to avoid issues associated with high-risk reagents (such as DAST), harsh conditions (cryogenic temperatures, high pressure), and transition-metal residues. This trend benefits from the increasing commercial availability of fluorinated building blocks and provides a clear direction for future process research and development of new CF_2_-containing drugs.

## Data Availability

Data sharing is not applicable as no new data was generated or analyzed in this study.
